# Multi‐Centre Reproducibility of DTI and NODDI in White Matter Tracts Segmented Using TractFinder Across Three MRI Scanners of the Same Model

**DOI:** 10.1002/hbm.70491

**Published:** 2026-03-30

**Authors:** Agnieszka Sierhej, Marta M. Correia, C. John Evans, Kiran K. Seunarine, Jonathan D. Clayden, Nadia A. S. Smith, Matt G. Hall, Chris A. Clark

**Affiliations:** ^1^ UCL GOS Institute of Child Health University College London London UK; ^2^ National Physical Laboratory Teddington UK; ^3^ Great Ormond Street Hospital for Children NHS Foundation Trust London UK; ^4^ MRC Cognition and Brain Sciences Unit University of Cambridge Cambridge UK; ^5^ CUBRIC, School of Psychology Cardiff University Cardiff UK; ^6^ TÜV SÜD UK Warrington UK; ^7^ Royal Surrey NHS Foundation Trust Guildford UK

**Keywords:** diffusion MRI, DTI, multi‐centre, NODDI, reproducibility, TractFinder, white matter

## Abstract

Quantitative imaging biomarkers (QIBs) are objective measures derived from quantitative imaging that can differentiate pathological changes from healthy biological processes. Diffusion MRI parameters derived from Diffusion Tensor Imaging (DTI) and Neurite Orientation Dispersion and Density Imaging (NODDI) could serve as potential QIBs for studying both healthy neurodevelopment and various neurological conditions. However, quantitative neuroimaging studies often require large datasets collected across multiple scanners, which introduces variability. To ensure the reliability of multi‐centre studies, the inter‐centre reproducibility of DTI and NODDI parameters must be thoroughly assessed before data collection begins. Discrepancies between results reported by previous studies can be explained by other sources of variability. The inter‐scanner reproducibility of diffusion parameters needs to be determined when the other sources of variability, such as differences in acquisition parameters, processing and ROI segmentation are controlled for. We assess the reproducibility of DTI and NODDI parameters in clinically relevant white matter (WM) tracts across three scanners of the same model, ensuring consistency in the acquisition scheme and pre‐processing pipelines. WM tract regions of interest (ROIs) are automatically segmented to standardise the analysis. Additionally, we investigate ROI and signal‐to‐noise ratio differences to better understand the sources of variability in diffusion parameters. According to the Koo and Li classification system, our results demonstrate excellent reproducibility for fractional anisotropy and mean diffusivity across scanners of the same model (ICC ≥ 0.964) when using identical acquisition schemes, pre‐processing pipelines and automated ROI segmentation. NODDI orientation dispersion index and neurite density index exhibit a similar level of reproducibility (ICC ≥ 0.942 and ICC ≥ 0.911, respectively), while free water fraction (FWF) has ICC ≥ 0.862. However, statistically significant variability was observed in the FWF, specifically within the left inferior fronto‐occipital fasciculus (CoV 9.43%) and optic radiation (CoV 9.95%), even when scanning the same cohort across sites. If there is an error in the signal fraction in one compartment in the NODDI model, the signal fractions from other compartments may likely be misestimated. The reproducibility and variability of diffusion parameters reported in this study provide guidance for future QIB research involving datasets derived from multiple scanners. These findings can help determine whether observed changes in diffusion parameters reflect meaningful biological differences or are highly influenced by measurement variability.

## Summary of the Key Message

– Determining multi‐centre reproducibility of DTI and NODDI parameters in healthy subjects provides reference values that are crucial in planning future multi‐centre studies.
– FA and MD have excellent reproducibility (ICC ≥ 0.964) across scanners of the same model when using identical acquisition schemes, pre‐processing pipelines and automatically segmented ROIs.
– NODDI ODI and NDI also have excellent reproducibility (ICC ≥ 0.911). However, caution is advised when using FWF, as statistically significant differences were detected in the left IFOF and OR.

**Abbreviations**
AF: arcuate fasciculus
CI: confidence interval
CoV: coefficient of variation
CSD: constrained spherical deconvolution
CSF: cerebro‐spinal fluid
CST: cortico‐spinal tract
DTI: diffusion tensor imaging
DWI: diffusion‐weighted imaging
EPI: echo planar imaging
FA: fractional anisotropy
FOD: fibre orientation distribution
FOV: field of view
FWF, V_iso_
: free water fraction
ICC: intra‐class corelation coefficient
IFOF: inferior fronto‐occipital fasciculus
MD: mean diffusivity
MPRAGE: magnetisation prepare rapid gradient echo
MRI: magnetic resonance imaging
NDI: neurite density imaging
NEMA: National Electrical Manufacturers Association
NODDI: neurite orientation dispersion and density imaging
ODI: orientation dispersion index
OR: optic radiation
PGSE: pulsed gradient spin echo
PSP: progressive supranuclear palsy
QIB: Quantitative imaging biomarker
ROI: region of interest
SD: standard deviation
SNR: signal‐to‐noise ratio
TE: echo time
TI: inversion time
TR: repetition time
WLS: weighted‐least squares
WM: white matter

## 1 Introduction

The ultimate goal of quantitative magnetic resonance imaging (MRI) is to objectively and non‐invasively support the diagnosis, monitoring and clinical prognosis of various diseases. While radiological signs (i.e., a recognisable feature in a clinical image) are common in neurological assessment, for example, hummingbird sign in progressive supranuclear palsy (PSP) (Gröschel et al. 2006), they rely on subjective evaluation and, thus, are subject to inter‐rater variability. Quantitative imaging biomarkers (QIBs) are objective measures derived from quantitative imaging that can differentiate pathological changes from healthy biological processes (Sullivan et al. 2015).

Diffusion tensor imaging (DTI) (Basser et al. 1994) is an established diffusion MRI technique to study microstructural changes in the brain, such as those occurring during normal neurodevelopment (Lebel et al. 2012). Neurite orientation dispersion and density imaging (NODDI) is a widely used multi‐compartment diffusion model aiming to provide more specificity than DTI in the characterisation of the underlying brain microstructure (Zhang et al. 2012). Parameters from these models have the potential to become QIBs for both healthy neurodevelopment and a variety of neurological conditions. It is important to determine the reproducibility of measured biomarkers and sources of uncertainty in QIBs prior to deploying them in clinical studies (O'Connor et al. 2017). Quantitative neuroimaging studies often require large datasets (Horien et al. 2021), collected across multiple scanners. Because of potential differences in gradient hardware, signal‐to‐noise ratio and other factors, the inter‐centre reproducibility of DTI and NODDI parameters should be determined before embarking on a multi‐centre study to ensure reliability of the reported results.

Reproducibility is defined as a measure of precision when the conditions of the measurements are different (e.g., different location, different operator, different measurement process), but the object of the measurement stays the same or similar (Sullivan et al. 2015). A commonly used metric to assess reproducibility is intra‐class correlation coefficient (ICC). Koo and Li (Koo and Li 2016) propose a classification system in which excellent reproducibility is defined if ICC > 0.9, moderate if ICC is between 0.75 and 0.9, moderate if ICC is between 0.5 and 0.75 and poor if ICC < 0.5. Moderate (ICC 0.5) (Helmer et al. 2016) to good (ICC 0.8–0.82) (Vollmar et al. 2010; Huang et al. 2012; Grech‐Sollars et al. 2015; Vavasour et al. 2019) reproducibility has been reported in frontal and global white matter (WM) regions of interest (ROIs) for DTI‐derived fractional anisotropy (FA). Reproducibility of FA in the corpus callosum also ranges from moderate (ICC 0.5–0.69) (Helmer et al. 2016; Huang et al. 2012; Grech‐Sollars et al. 2015; Vavasour et al. 2019) to good (ICC 0.8–0.9) (Vollmar et al. 2010; Grech‐Sollars et al. 2015; Vavasour et al. 2019). In other WM tracts (cortico‐spinal tract (CST), uncinate Fasciculus, superior longitudinal fasciculus), the reproducibility of FA ranges from poor to excellent (ICC 0.32–0.94) (Vollmar et al. 2010; Huang et al. 2012; Grech‐Sollars et al. 2015; Deprez et al. 2018; Zhou et al. 2018). The discrepancies between reported results can be explained by other sources of variability, including differences in WM tract segmentation protocols (Schilling et al. 2021). Intra‐scanner variability of NODDI parameters has been assessed previously (Chung et al. 2016; Lehmann et al. 2021; Lucignani et al. 2021; Mueller et al. 2023). Low variability is reported in both the orientation dispersion index (ODI) and neurite density index (NDI) in major WM tracts (coefficient of variation (CoV) < 4%), while free water fraction (FWF or *V*_iso_) has large variability (CoV > 10%) (Lehmann et al. 2021; Mueller et al. 2023). Inter‐scanner reproducibility of NODDI parameters has been studied across different field strengths (Chung et al. 2016) and vendors (Andica et al. 2020). In CST, NODDI parameters exhibit generally poor reproducibility across scanners from different vendors (Andica et al. 2020). However, the use of different head coils (32‐channel and 64‐channel) on two of the scanners introduces additional variability beyond the vendor‐related effects (Panman et al. 2019). The inter‐centre reproducibility of diffusion parameters needs to be determined when the other sources of variability, such as differences in acquisition parameters, processing, and ROI segmentation are controlled for.

WM ROIs are often determined using streamline tractography (Schilling et al. 2021), allowing diffusion parameters in specific WM tracts to be characterised. A key issue with streamline tractography is the lack of reproducibility, as it is fundamentally user dependent process, highly dependent on individual preferences (Schilling et al. 2021). Automated segmentation approaches eliminate intra‐rater variability and thus, are more suitable for reproducibility studies (Clayden et al. 2009). The recently introduced TractFinder (Young et al. 2024) method is a technique for streamline‐free WM tract segmentation, which incorporates both spatial and orientational priors. The main advantage of TractFinder over other automatic techniques that rely on deep‐learning tools, for example, TractSeg (Wasserthal et al. 2018), is that it produces consistent results rapidly, and has a fully explainable algorithm (Young et al. 2024). TractFinder uses a simple mathematical formulation combining spatial and orientational atlases with the subject's native data, which allows for an intuitive understanding of why a given voxel is included in the tract segmentation. A further advantage of TractFinder is of its rapid completion time (< 3 min running on an iMac with 3.8 GHz Quad‐Core Intel Core i5). TractFinder is fully compatible with segmenting WM tracts from healthy subjects' datasets, and also has application in areas such as surgical planning.

The aim of this study is to assess the reproducibility of DTI‐derived and NODDI‐derived parameters in clinically relevant WM tracts across three scanners of the same model, ensuring identical acquisition scheme and pre‐processing pipelines, and where the WM tract ROIs are automatically segmented. It is crucial to isolate and understand inter‐scanner variability from the same MRI scanner model before trying to understand more complex multi‐vendor combinations. Additional analysis of ROI and signal‐to‐noise ratio (SNR) variability is carried out to better understand the sources of variation in diffusion parameters. The reproducibility results from this study are therefore designed to inform future multi‐centre neuroimaging studies in which the consortium members are using MRI hardware of the same model and vendor.

## 2 Materials and Methods

### 2.1 Volunteers

The data for this study was acquired across three centres: UCL GOS Institute of Child Health and Great Ormond Street Hospital for Children (datasets labelled UCL), MRC Cognition and Brain Sciences Unit, University of Cambridge (datasets labelled CAM) and CUBRIC, School of Psychology, Cardiff University (datasets labelled CAR). Ethical approval (number 2780/001) for this study was granted by the UCL Research Ethics Committee (REC). In each case, scanner hardware was as described in Section 2.2 below.

Ten healthy adult volunteers with no diagnosis of any neurological conditions underwent advanced diffusion MRI brain scans between December 2017 and September 2018 at each of the three scanners. All volunteers were subjected to a health check questionnaire and metal checklist before the scan to ensure that they did not have any contraindications to the MRI scan. Informed signed consent was obtained from all volunteers so that the data acquired could be used for research purposes. Cohort details are summarised in Table 1. Acquisition order was randomised between the volunteers to eliminate bias due to order effects.

**TABLE 1:** The cohort information (identical for each of the scanners).

Cohort information	
Age in years at the first scan (mean ± SD) [range]	36.1 ± 12.05 [25–58]
Number of volunteers (female:male)	5:5
Time between first and second scan in days (mean ± SD)	101 ± 52
Time between second and third scan in days (mean ± SD)	56 ± 30

### 2.2 Data Acquisition

Diffusion and T_1_‐weighted images were acquired on three Siemens MAGNETOM Prisma 3 T scanners (Siemens Healthcare, Erlangen, Germany) with a maximum gradient strength of 80 mT/m, maximum slew rate of 200 T/m/s and using a 20‐channel head–neck receive coil. The choice of 20‐channel head–neck receive coil over the 64‐channel head–neck receive coil was motivated by the potential of clinical translation. 64‐channel head–neck receive coil has better overall SNR in cortical areas when compared to 20‐channel head–neck receive coil, and it is commonly used to acquire functional MRI data in research studies. However, in the clinical settings, the 20‐channel head–neck receive coil is used more often. Therefore, this dataset was acquired with the 20‐channel head–neck receive coil, because it represents the real‐life clinical situation and supports the clinical translation. The software versions were Syngo MR D13D at the Cambridge site, Syngo MR E11X at the Cardiff site, and Syngo MR D13D at the UCL site. The T_1_‐weighted image was acquired using a 3D Magnetisation‐Prepared Rapid Gradient Echo (MPRAGE) sequence (Mugler and Brookeman 1991) with coronal orientation. Diffusion‐weighted images (DWI) were acquired using pulsed gradient spin echo (PGSE) with echo planar imaging (EPI) readout with anterior–posterior (A‐P) phase encoding. Sixty gradient directions (with identical directional point sets at each site) per *b* value (*b* = 1000, 2200 s/mm^2^) were acquired, in addition to 13 unweighted images (*b* = 0 s/mm^2^). The gradient pulse duration time was 16.69 ms, while the gradient pulse separation time was 28.71 ms. Other factors include GRAPPA *R* = 2 (The Minnesota multiband EPI sequence with multiband (MB) factor 2), and partial Fourier was set to 6/8. Additionally, one unweighted image (*b* = 0 s/mm^2^) in the reversed‐phase encoding (P > A) was acquired to be used in distortion correction. Other acquisition parameters are included in Table 2. Total scanning time was 13 min 25 s (including localizer) per session.

**TABLE 2:** The scan parameters (identical for each of the scanners).

Acquisition Parameters			
	T_1_	DWI	Reversed‐phase encoding image
TR/TE (ms)	2300/2.74	3050/60	3050/60
FOV	256 × 256	220 × 220	220 × 220
Voxel dimensions (mm)	1 × 1 × 1	2 × 2 × 2.2	2 × 2 × 2.2
Number of slices	240	66	66
TI (ms)	909	—	—
Flip angle	8	90	90
Time	5 min 21 s	7 min 16 s	0 min 34 s

Abbreviations: DWI, diffusion‐weighted imaging acquisition; FOV, field of view; T_1_, T_1_‐weighted acquisition; TE, echo time; TI, inversion time; TR, repetition time.

### 2.3 Pre‐Processing Pipeline

The images were pre‐processed using a pipeline in MRtrix3 (Tournier et al. 2019). Firstly, the images were denoised using an estimation of noise levels via a Marchenko–Pastur principal component analysis (Veraart et al. 2016). Then, Gibbs ringing artefacts were removed with the local sub‐voxel shifts method (Kellner et al. 2016). Inhomogeneity distortions, motion and eddy current artefacts were corrected using MRtrix3's (Tournier et al. 2019) implementation of FSL's (Jenkinson et al. 2012) eddy (which includes the use of FSL topup using reversed‐phase encode unweighted images). Lastly, the bias induced by the B_1_ field inhomogeneity correction was performed with the FSL algorithm (Jenkinson et al. 2012) implemented in MRtrix3 (Tournier et al. 2019).

### 2.4 DTI and NODDI Fitting

DTI‐derived FA and Mean Diffusivity (MD) maps were fitted using FSL's ‘dtifit’ (Jenkinson et al. 2012). DTI was fitted using a full dataset to avoid bias due to a decreased number of gradient directions when compared to NODDI results. A weighted‐least squares (WLS) fitting was used to minimise the contribution of the noise present in the shell with a higher b‐value to the DTI fit. FA and MD maps are estimated using a set of eigenvalues in each voxel (Basser et al. 1994). The NODDI model was fitted with MATLAB's toolbox (version 1.0.5) (Zhang et al. 2012). We derived the following NODDI parameter maps: FWF—free water fraction, also known as volume fraction of the CSF compartment or isotropic volume fraction (unitless); NDI—neurite density index, also known as intra‐cellular volume fraction (unitless). ODI—orientation dispersion index (OD in the original NODDI paper (Zhang et al. 2012)); ODI ranges from 0, for perfectly aligned fibres, to 1, for isotropic distribution of fibres (unitless) (Zhang et al. 2012).

### 2.5 Regions of Interest (ROIs)

Following the registration of the T_1_‐weighted images to the diffusion space using the NiftyReg aladin algorithm (Modat et al. 2010; Ourselin et al. 2001), TractFinder (Young et al. 2024) was used to segment WM tracts: CST, arcuate fasciculus (AF), optic radiation (OR) and inferior fronto‐occipital fasciculus (IFOF). These four WM tracts have substantial clinical relevance. CST is involved in voluntary motor function, such as hand coordination. Functions of CST have been extensively studied in the context of abnormal conditions, for example, after stroke (Lo et al. 2010). AF may have an important role in reading skills and language functions (Vandermosten et al. 2012; Yeatman et al. 2011), while OR mainly supports vision (Bullock et al. 2022). IFOF is thought to be involved in semantic language processing (Conner et al. 2018). To limit contamination from non‐WM tissue, the final ROIs were the product of a binary multiplication of TractFinder (Young et al. 2024) tracts and a WM mask. TractFinder atlases were designed with the idea to extend well into GM to ensure the full coverage of WM tract. Therefore, it was essential to limit the coverage of the tract to avoid contamination of the ROI with voxels from GM and CSF. The WM mask was estimated with fibre orientation distributions (FODs) from diffusion data using multi‐shell multi‐tissue constrained spherical deconvolution (CSD) (Tournier et al. 2019; Jeurissen et al. 2014) and thresholded at 0.2, that is, the voxels with volume fraction above 0.2 were included. The threshold of 0.2 was chosen based on the visual inspection of segmented WM ROI. Final WM ROIs were estimated by multiplication of full extent TractFinder segmentation with binary WM mask derived from msmtCSD. To obtain the volumes of specific tracts, first, the resulting ROIs were converted into binary masks. Then, the number of voxels in a given tract was multiplied by the volume of the voxel (see voxel dimension of DWI in Table 2).

### 2.6 Reproducibility and Variability Assessment

Mean values of diffusion parameters (FA, MD, ODI, NDI, FWF) in each ROI were calculated for each subject and each scanner and plotted in the form of boxplots. Reproducibility between the scanners was assessed using a two‐way mixed effect model ICC (Koo and Li 2016), defined in the following equation:

(1) $$\mathrm{ICC}=\frac{\sigma_{B}^{2}}{\sigma_{B}^{2}+\frac{\sigma_{E}^{2}}{k}}$$

where $\sigma_{B}^{2}$ is the between‐subject variance, $\sigma_{E}^{2}$ is the within‐subject variance (error variance) and *k* is the number of scanners.

Single‐factor ANOVA (Laverty and Kelly 2019) was performed in MS Excel to verify the presence of significant differences between results acquired on different scanners. Statistical significance was set to conventional *p* < 0.05. We report the effect size (𝜂^2^) (Maher et al. 2013) alongside the *p* values. We did not perform multiple comparison correction to avoid artificially lowering the level of statistical significance which could lead to an increase rate of Type II errors. We use the coefficient of variation (CoV) (Equation 2) to assess how the mean and standard deviation (SD) of the diffusion parameters in each ROI vary between the three scanners. This is defined as

(2) $$\begin{array}{c} \mathrm{CoV}=\frac{\sigma }{\mu }\times 100\% \end{array}$$

where σ is the SD of the diffusion parameter in each ROI from three scanners and μ is the mean of the diffusion parameter in each ROI from three scanners.

Lehman et al. (Lehmann et al. 2021) suggest that SNR is important in determining NODDI and DTI parameter reproducibility. To further explain variability in diffusion parameters, the potential differences in ROIs and SNRs were assessed. In both cases, single‐factor ANOVA (Laverty and Kelly 2019) was performed to verify the presence of significant differences between tract volumes segmented from data acquired on different scanners and between global SNR and ROI‐specific SNR from data acquired on different scanners. We calculated the SNR using NEMA Method 4 (Maher et al. 2013). The correction factor was set to 0.7 for a 20‐channel head–neck coil. We report ICC of WM tract volumes and SNR between three scanners. CoV of WM tract volumes and SNR between the scanners for each volunteer is calculated. Then, the mean CoV is reported along with the 95% confidence interval (CI).

## 3 Results

**FIGURE 1:**
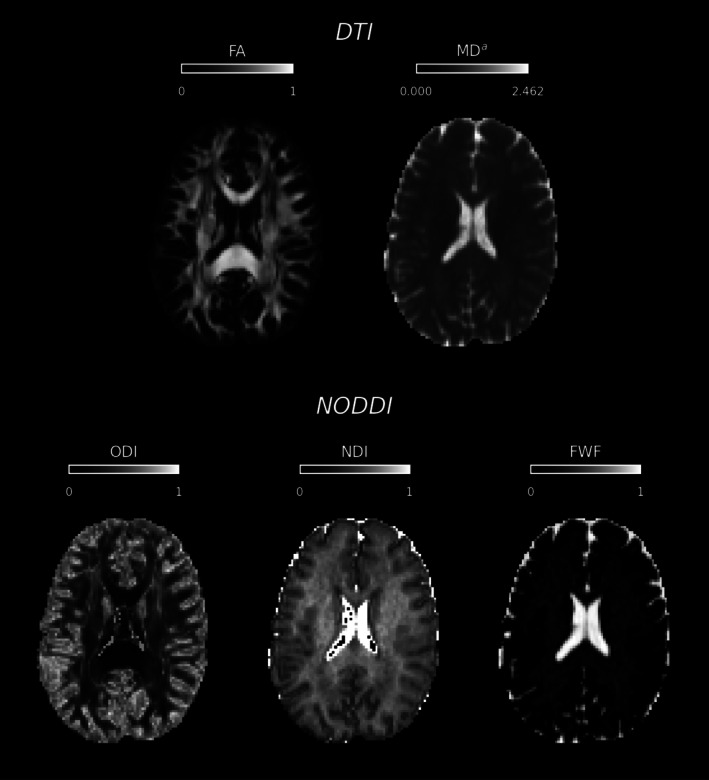
Example parameter maps for DTI (top row) and NODDI (bottom row). ^a^Unit: $\times 10^{-3}\frac{{\mathrm{mm}}^{2}}{s}$.

**FIGURE 2:**
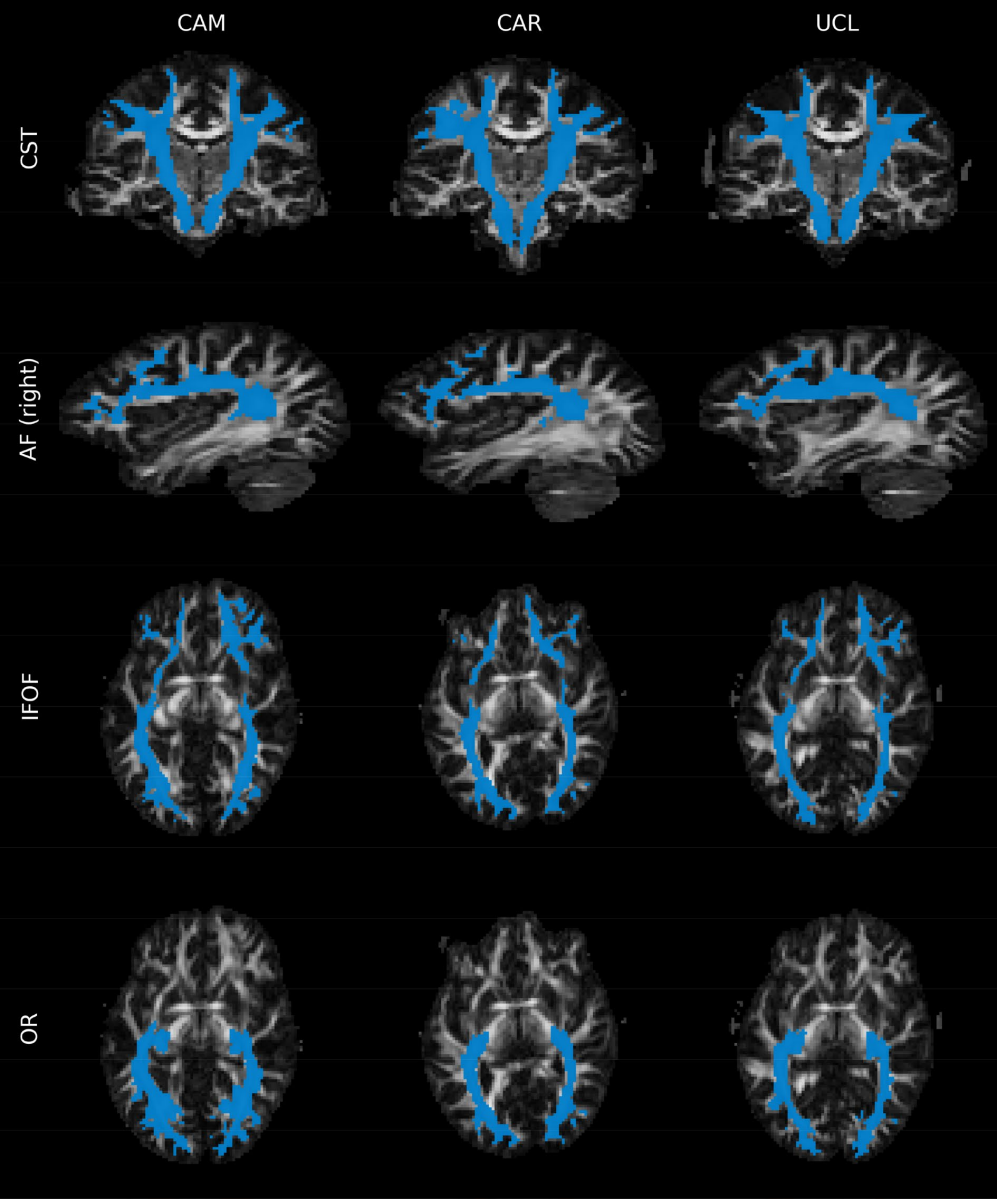
Visual comparison of WM tracts between the centres for a single subject. ROIs are shown in blue overlayed on top of representative slices of an FA map. CST: coronal slice (CAM, CAR, UCL: 54/110); AF (right): sagittal slice (CAM: 72/110; CAR: 73/110; UCL: 71/110); IFOF: axial slice (CAM: 27/66; CAR: 28/66; UCL: 27/66); OR: axial slice (CAM: 27/66; CAR: 28/66; UCL: 27/66).

Example DTI and NODDI parameter maps are shown in Figure 1. Low FA can be found in the WM regions where crossing fibres are present. Spurious ODI values can be found in the voxels belonging to the ventricles. A similar occurrence can be observed for NDI, which takes the value of 1 (signal fraction is estimated to be originating entirely from the intra‐cellular compartment) in the cerebro‐spinal fluid (CSF) filled voxels. The positioning of the ROIs generated using TractFinder (Young et al. 2024) within the brain is visualised in Figure 2 for a representative dataset and across three scanners.

Table 3 gives ICC values for diffusion parameters in the WM tracts considered. DTI parameters have excellent reproducibility (Koo and Li 2016) in all ROIs (ICC > 0.964). Similarly, NODDI‐derived ODI and NDI have ICC > 0.911. FWF has excellent reproducibility in all but two ROIs. In the left and right CST, ICC for FWF is 0.885 and 0.862, respectively, reducing the level of reproducibility to good (Koo and Li 2016). ANOVA analysis (Table 4) reveals statistically significant differences in FWF in left IFOF and OR. All other parameters have no statistically significant differences between the centres.

**TABLE 3:** ICC for the mean DTI and NODDI parameters in WM tracts across three scanners of the same model.

	ICC							
	Left				Right			
	CST	AF	IFOF	OR	CST	AF	IFOF	OR
DTI								
FA	**0.991**	**0.985**	**0.977**	**0.973**	**0.986**	**0.995**	**0.991**	**0.983**
MD	**0.964**	**0.986**	**0.969**	**0.968**	**0.974**	**0.974**	**0.979**	**0.975**
NODDI								
ODI	**0.975**	**0.970**	**0.956**	**0.942**	**0.978**	**0.992**	**0.988**	**0.972**
NDI	**0.911**	**0.948**	**0.933**	**0.926**	**0.929**	**0.992**	**0.961**	**0.958**
FWF	0.885	**0.939**	**0.909**	**0.910**	0.862	**0.908**	**0.932**	**0.941**

*Note:* Excellent reproducibility (Koo and Li classification (Koo and Li 2016)) is marked in bold.

**TABLE 4:** *p* values from ANOVA analysis.

	ANOVA *p* value							
	Left				Right			
	CST	AF	IFOF	OR	CST	AF	IFOF	OR
DTI								
FA	0.65 (0.031)	0.92 (0.006)	0.86 (0.012)	0.85 (0.012)	0.76 (0.020)	0.97 (0.003)	0.98 (0.002)	0.95 (0.004)
MD	0.28 (0.091)	0.60 (0.037)	0.51 (0.048)	0.51 (0.049)	0.12 (0.147)	0.64 (0.032)	0.70 (0.026)	0.63 (0.033)
NODDI								
ODI	0.49 (0.052)	0.80 (0.017)	0.76 (0.020)	0.74 (0.023)	0.49 (0.052)	0.87 (0.010)	0.98 (0.002)	0.94 (0.004)
NDI	0.80 (0.016)	0.85 (0.012)	0.61 (0.036)	0.71 (0.025)	0.63 (0.033)	0.74 (0.022)	0.57 (0.041)	0.52 (0.047)
FWF	0.18 (0.120)	0.12 (0.146)	0.03* (0.231)	0.04* (0.208)	0.16 (0.128)	0.27 (0.094)	0.08 (0.174)	0.11 (0.153)

*Note:* *p* values in bold and with * indicates statistically significance (conventional *p* < 0.05). The effect size (*𝜂* ^2^) is given alongside the *p* value in brackets.

**FIGURE 3:**
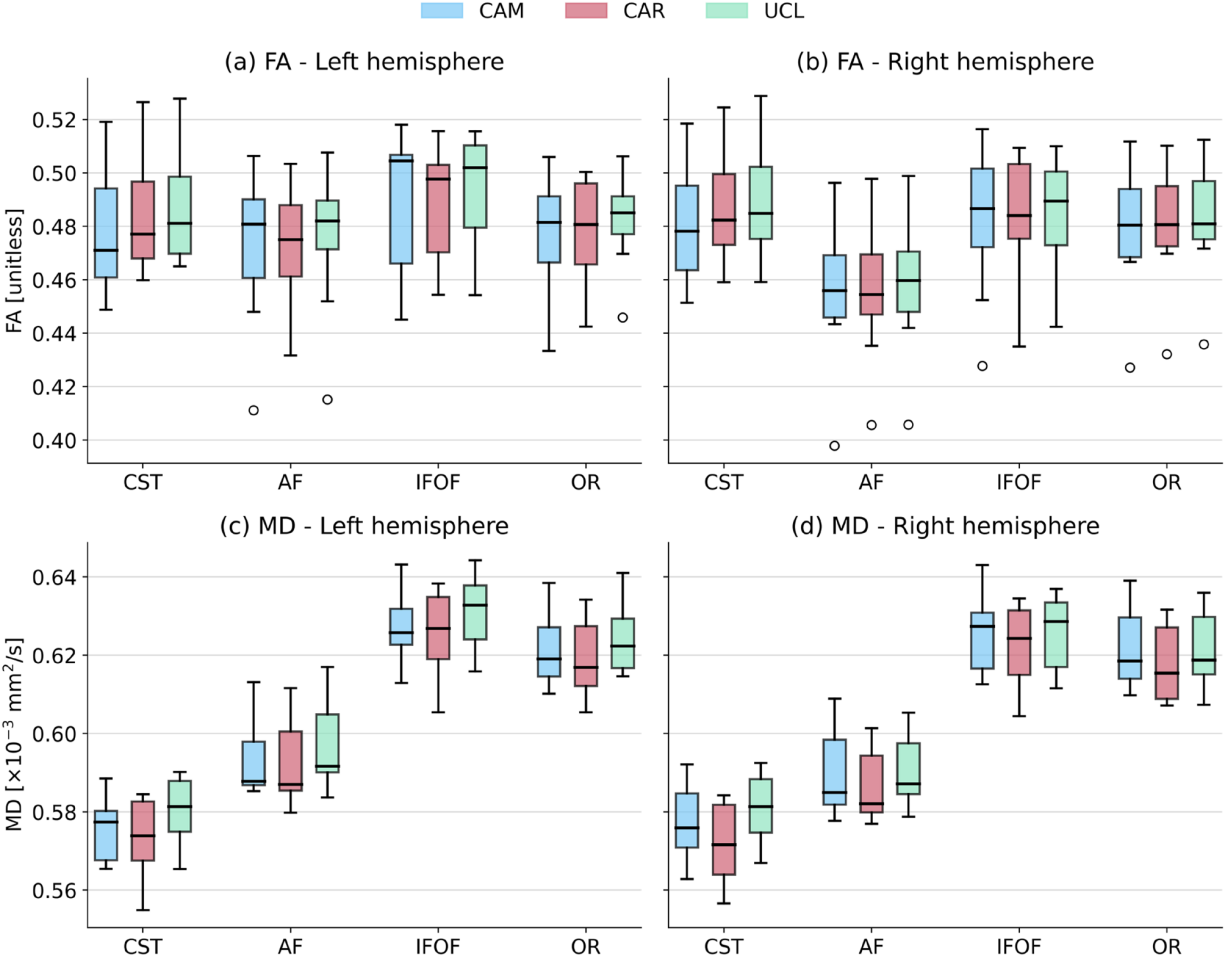
Variability of FA and MD in WM tracts across the three sites. No significant differences between parameters from the three sites were found. CAM, CAR and UCL are labels of scanners (from their site names). Boxes represent the inter‐quartile range. Whiskers indicate the farthest data point lying within 1.5 times the inter‐quartile range from the box. Fliers (°) represent outliers.

**FIGURE 4:**
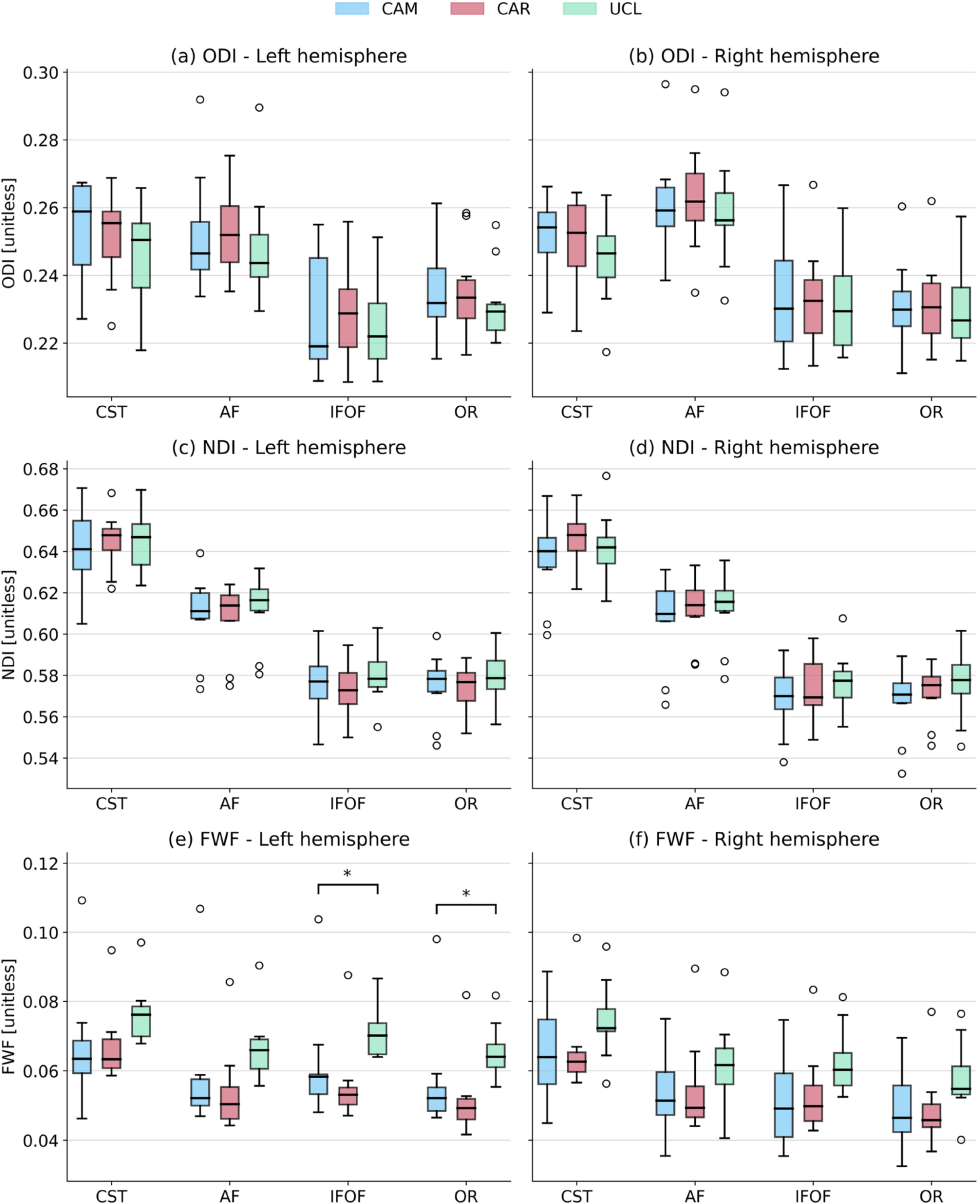
Variability of ODI, NDI and FWF in WM tracts across the three sites. CAM, CAR and UCL are labels of scanners (from their site names). Boxes represent the inter‐quartile range. Whiskers indicate the farthest data point lying within 1.5 times the inter‐quartile range from the box. Fliers (°) represent outliers. (*) marks significant difference between the results from different scanners (from ANOVA).

The variability of DTI parameters in both the left and right hemispheres is shown in Figure 3. No significant differences between the results from different scanners were found in any of the ROIs. Figure 4 shows boxplots for NODDI parameters. There are no statistically significant differences between the centres for ODI and NDI (Figure 4). Significant differences are present in FWF in the left IFOF and OR (Figure 4).

Table 5 shows the variation of the mean and SD of DTI and NODDI parameters between scanners in each ROI. Low CoV of group mean across three scanners was present in both FA and MD (0.17%–0.78% and 0.27%–0.64%, respectively). The CoV of group SD across the scanners was also low in FA (0.32%–0.84%) and slightly higher in MD (0.85%–2.28%). Both ODI and NDI had low CoV of group mean (0.2%–1.17% and 0.28%–0.64%, respectively). The CoV of group SD in ODI was 0.46%–1.13% and higher in NDI (0.76%–3.39%). The CoV of group mean in FWF was higher than other parameters (6.06%–9.95%). The CoV of group mean of the parameter values across three scanners in left OR was 9.95% and in left IFOF 9.43%, which is reflected by statistical significance found by ANOVA testing in these ROIs. Similarly, group SD in FWF had a higher CoV compared to other parameters (1.98%–4.88%).

**TABLE 5:** Variation (CoV) of mean and SD across three scanners for DTI and NODDI parameters.

	Left				Right			
	CST	AF	IFOF	OR	CST	AF	IFOF	OR
DTI FA								
Mean	0.78%	0.43%	0.45%	0.53%	0.61%	0.31%	0.26%	0.17%
SD	0.32%	0.39%	0.36%	0.33%	0.84%	0.41%	0.67%	0.38%
DTI MD^a^								
Mean	0.50%	0.36%	0.33%	0.39%	0.64%	0.32%	0.27%	0.27%
SD	1.11%	1.29%	2.13%	1.43%	1.06%	1.36%	2.28%	0.85%
NODDI ODI								
Mean	1.17%	0.68%	0.81%	0.95%	1.06%	0.63%	0.35%	0.20%
SD	0.76%	1.04%	0.46%	0.72%	0.87%	0.53%	0.50%	1.13%
NODDI NDI								
Mean	0.29%	0.28%	0.38%	0.51%	0.45%	0.41%	0.64%	0.50%
SD	2.50%	1.48%	2.67%	2.44%	3.39%	0.76%	2.83%	2.21%
NODDI FWF								
Mean	6.06%	9.11%	9.43%	9.95%	6.24%	6.24%	8.70%	9.11%
SD	2.03%	3.47%	4.06%	4.88%	2.80%	2.11%	1.98%	2.25%

^a^ Unit: $\times 10^{-3}\frac{{\mathrm{mm}}^{2}}{s}$.

No statistically significant difference was found between the tract volumes segmented from data acquired with different scanners (the lowest *p* value was 0.94 –Table 6). Similarly, the differences in SNR between different scanners were also not statistically significant in both whole‐brain SNR (*p* = 0.8) and all WM ROIs considered (*p* > 0.71—Table 6).

**TABLE 6:** *p* values from ANOVA analysis for tract volumes and tract SNR.

		Left				Right			
		CST	AF	IFOF	OR	CST	AF	IFOF	OR
Tract volume	ANOVA *p* value (*𝜂* ^2^)	0.98 (0.002)	0.99 (0.001)	0.98 (0.002)	0.94 (0.005)	0.98 (0.001)	0.98 (0.001)	0.98 (0.002)	0.98 (0.002)
	ICC	0.984	0.994	0.968	0.990	0.995	0.993	0.992	0.995
	Mean CoV [95% CI]	3.15% [2.98%, 3.32%]	2.41% [2.26%, 2.56%]	4.99% [4.71%, 5.27%]	4.10% [3.79%, 4.41%]	2.13% [2.01%, 2.25%]	2.51% [2.33%, 2.69%]	3.27% [3.03%, 3.51%]	3.64% [3.30%, 3.98%]
SNR in the ROI	ANOVA *p* value (*𝜂* ^2^)	1.00 (< 0.001)	0.89 (0.009)	0.72 (0.024)	0.93 (0.006)	0.87 (0.010)	0.79 (0.018)	0.36 (0.072)	0.71 (0.026)

*Note:* No statistical significance was found between the three scanners. The effect size (*𝜂* ^2^) is given alongside the *p* value in brackets. ICC metric and mean CoV between the scanners with 95% CI are provided for tract volumes.

## 4 Discussion

Quantitative neuroimaging studies aiming to establish QIBs for healthy neurodevelopment as well as neurological conditions often require large amounts of data, so the results can be generalisable on a population level (National Electrical Manufacturers Association 2014). Therefore, it is common practice to collect the data using multiple scanners from different sites. To ensure that the detected change in the MRI‐derived parameter is meaningful and can be established as a QIB, it is critical to disentangle measurement variability (intra and inter‐scanner effects) from true inter‐individual biological variation. Our study addresses this by evaluating the reproducibility and variability of DTI and NODDI parameters across three identical scanners, using an identical acquisition protocol, pre‐processing pipeline and consistent ROI segmentation.

As shown in Figures 3 and 4, DTI parameters exhibited comparable median and inter‐quartile ranges across scanners, suggesting that observed variability present in these parameters primarily reflects inter‐individual variability rather than inter‐scanner differences. In contrast, NODDI parameters demonstrated larger inter‐scanner disparities in both median values and inter‐quartile ranges, indicating a stronger influence of the inter‐scanner effects.

Our study demonstrates excellent reproducibility (Koo and Li 2016) of DTI parameters (ICC ≥ 0.964 across all ROIs, see Table 3), surpassing variability ranges reported in prior multi‐scanner investigations for several WM regions (ICC = 0.32–0.94) (Helmer et al. 2016; Vollmar et al. 2010; Huang et al. 2012; Grech‐Sollars et al. 2015; Vavasour et al. 2019; Deprez et al. 2018; Zhou et al. 2018). This discrepancy likely stems from methodological differences, for example, variability from manually segmented ROIs (Schilling et al. 2021), differences in acquisition schemes, different field strengths and differences between vendors (e.g., image reconstruction algorithms). Our study uses an automatic and explainable algorithm, TractFinder (Young et al. 2024), to define ROIs (see Figure 2), eliminating inter‐rater variability. ANOVA confirmed no statistically significant inter‐scanner differences in ROI volumes, further indicating that differences in ROI definition may explain the large discrepancies and low reproducibility reported by previous studies.

While DTI remains a well‐established model in neuroimaging research (O'Connor et al. 2017; Assaf and Pasternak 2008; Alexander et al. 2007; Oishi et al. 2011; Tae et al. 2018; Gross 2011), its reliance on the single‐tensor model introduces well‐documented limitations in regions with complex fibre geometries. As shown in Figure 1, crossing fibres in the WM region artificially reduce the FA values, uncharacteristic of what would otherwise be expected from healthy WM fibres. By introducing ODI, NODDI attempts to provide more specific information about fibre geometries inside a given voxel compared to FA from DTI.

NODDI‐derived ODI and NDI parameters demonstrate excellent inter‐scanner reproducibility (Koo and Li 2016) (ICC > 0.911), consistent with previous studies that reported good scan‐rescan repeatability in these parameters (Lehmann et al. 2021; Mueller et al. 2023). FWF has excellent reproducibility in all but one ROI. In the left CST, ICC for FWF is 0.885, reducing the level of reproducibility to good, according to Koo and Li classification (Koo and Li 2016). ANOVA analysis (Table 4) reveals statistically significant differences in FWF in left IFOF and OR. Previous studies reported larger variability in FWF (CoV > 10%) (Lehmann et al. 2021; Mueller et al. 2023) when compared to other diffusion parameters. This is also shown in our analysis (Table 5). All other parameters show no statistically significant differences across centres.

NODDI's ODI assumes a Watson distribution to quantify axon bending and fanning in WM (Zhang and Burock 2020), but this cylindrical symmetry constraint limits accuracy in regions with crossing fibres or asymmetric dispersion patterns (Zhang and Burock 2020). Caution is advised when interpreting parameter maps fitted by such models (Zhang et al. 2012). Spurious ODI and NDI values in voxels that are present in ventricles, and therefore filled with CSF, indicate that the fit of the model should be treated with caution in these regions. Whilst it is clear the NODDI parameter values are spurious in regions such as ventricles, however, the extent of spurious results in the WM and GM regions is unknown. Jelescu et al. (Kamiya et al. 2020) uncovered a major problem with the bimodality of the multi‐compartment model fitting, making NODDI's results highly dependent on initialisation parameters and sensitive to noise. Lehman et al. (Lehmann et al. 2021) suggested that SNR is important in determining NODDI and DTI parameter reproducibility. To investigate potential confounding factors, we conducted supplementary ANOVA to assess whether SNR variability influenced observed differences in diffusion parameters across scanners. No statistically significant inter‐scanner SNR differences were detected across the three systems. This finding suggests that further analysis may be needed to explain the NODDI parameter variability. Mueller et al. (Mueller et al. 2023) speculate that variability in FWF may be explained in large turnover of volume exchange of extracellular fluid; however, further evidence‐based studies need to be conducted to evaluate this hypothesis.

Another aspect to consider is that ICC may not be enough on its own as a metric to characterise the inter‐scanner reproducibility in a trustworthy manner. Our results show that all diffusion parameters considered in this study have excellent reproducibility (ICC ≥ 0.908), according to Koo and Li classification (Koo and Li 2016), in all but two ROIs. FWF has lower reproducibility in the left and right CST (ICC 0.885 and ICC 0.862, respectively). However, ANOVA results show statistically significant differences between scanners for FWF in left IFOF and OR. This may be explained by the large between‐subject variance found in this parameter (See Table A2). Furthermore, the group SD varies quite significantly between scanners (CoV > 9%) for this parameter in both left IFOF and OR. Our analysis suggests that the conventional hypothesis testing frameworks provide critical validation of ICC‐based reproducibility claims. A high ICC (> 0.9) ensures clinically reliable parameter reproducibility if inter‐scanner measurements for identical cohorts do not show significant mean differences, where expectation is that no significant differences should exist. In this study, we run single‐factor ANOVA analysis alongside conventional reproducibility metric (ICC) to verify whether there are no statistically significant differences even when ICC is high (> 0.9). It is important to note and discuss that we did not use multiple comparison corrections in the statistical analysis. While multiple comparison correction is a widely accepted method to control for false positives in statistical analysis, it dramatically reduces statistical power and the ability to detect true effects. Applying stringent corrections could lead to Type II errors, where real effects are incorrectly deemed insignificant (Table A1).

While FWF may not serve as a standalone QIB, it is still important to assess its variability. As FWF is the volume fraction of isotropic compartment (see Equation 2), systematic errors in its estimation can propagate to other compartments. This can directly impact the estimation of NDI, which is a volume fraction of the intra‐cellular compartment, as the error in volume fraction of one compartment may lead to misestimation of volume fractions of other compartments. For example, the tissue‐weighted mean technique (Jelescu et al. 2016), which is designed to minimise estimation bias in NODDI parameters when an ROI‐based analysis is involved, is highly dependent on the correct estimation of signal fractions. The rationale behind the technique is that not all voxels are equal, that is, the fraction of the voxel where the intra‐cellular compartment is present should be considered when computing the mean of NODDI parameters in each ROI. The fraction of the CSF (isotropic) compartment (FWF) estimated by NODDI is used in computing the weights (see Equation 2). As shown in this work and in a previous study (Chung et al. 2016), the variability of the group mean and observed inter‐individual SD of FWF is large across scanners of the same model and field strength, respectively, which puts in question reasonable use of FWF if the data is acquired from different scanners.

In the case of quantitative neuroimaging studies, one must carefully design the study to make sure that if the change between the control and studied cohort is detected, such change a is biologically meaningful. The level of reproducibility and variability of diffusion parameters reported here can inform future multi‐centre studies in which the consortium members use MRI scanners of the same model. If we control for acquisition protocol, processing pipeline and ROI segmentation variability, the reproducibility of DTI parameters is excellent (ICC > 0.9 (Koo and Li 2016)) and the reproducibility of NODDI parameters is good to excellent (ICC > 0.75 (Koo and Li 2016)). However, as shown by the previous study (Andica et al. 2020), introducing different vendors and different hardware setups (e.g., different coils) will only lower the reproducibility.

The current study assesses the inter‐scanner reproducibility and variability of diffusion parameters across three scanners of the same vendor and model, when variations originating from the differences in the acquisition protocol, processing steps and ROI segmentation are controlled for. However, we are aware that the reproducibility of diffusion parameters needs to be studied further across other sources of variability. Since many neuroimaging studies use different combinations of vendors and models of MRI scanners, this introduces non‐biological, that is, inter‐scanner and inter‐vendor, variability to the results. This non‐biological variability is unique for a given combination of vendors and models, so that the results of such study may vary from another study acquired with different combinations of vendors and models. Inter‐scanner reproducibility has been studied in the past and the results of reproducibility studies contain large discrepancies. Therefore, the current state of knowledge is that different vendors and different models bring non‐biological variability to the results, but this variability is not understood very well and needs to be characterised. The current study isolates inter‐scanner variability for a single model of MRI scanners and proposes a framework of systematically studying the variability and reproducibility for other vendors and models. This study's results can be used by future studies to distinguish whether the variability that they report in their study is biologically meaningful and to what extent it is due to the scanner variability. We do not claim that the current study gives all the information on how every single vendor and model adds to the observed variability. However, we emphasise how important it is to understand the variability in the scanners of the same model, before trying to understand more complex combinations, that is, different vendors. Inter‐vendor variability is an important aspect to assess as many multi‐centre studies are constrained by the practicality that the data is acquired at scanners across different models and vendors. Differences in image reconstruction algorithms between vendors are likely a large contribution to non‐biological variability (Parker et al. 2021). Hardware imperfections and differences (e.g., the use of head coils with different numbers of channels) may play an important role in increasing non‐biological variability. For example, a previous study found that in CST, NODDI parameters had poor reproducibility across scanners from different vendors (Andica et al. 2020), however, two scanners used different head coils, further contributing to variability. Field inhomogeneity and differences in SNR are also factors to consider. MB acquisition enables high angular resolution protocols with fast acquisition time, crucial in diffusion MRI acquisition (Karakuzu et al. 2022). The challenges include RF inhomogeneity and high SAR levels (Wu and Miller 2017), which may result in patient safety concerns. Physiological noise, partial volume effect and patient positioning will affect the variability. These can be minimised, for example, by using cardiac gating (Auerbach et al. 2013). However, in a clinical setup, these can be impractical to control for (except for cardiac gating), and thus when defining confidence intervals for QIBs, these should be included in the confidence intervals. Future work should address the analysis of the contribution of other sources of variability so they can be corrected, allowing the observed variability to reflect true inter‐individual variability with greater accuracy.

Moreover, we acknowledge that the current design may limit the generalisability of the results as other vendors may not have the same reproducibility. However, it is important to isolate inter‐scanner variability from the same model before trying to understand more complex cases, that is, when we have multiple models and multiple vendors. It is not feasible to analyse the multi‐vendor data in this current study, as this was not the aim of this study and therefore the design of the study did not include the data acquisition across multiple vendors. However, we recommend future studies to use the presented framework, and methods design to assess the reproducibility in other combinations of models and vendor with increasing complexity. In this way, the future studies can be informed about specific sources of variability rather than mixed vendor‐model sources, resulting in lack of understanding where the variability comes from, and thus, such results not being potentially generalisable to other similar studies which use different combinations of vendors‐models.

We use identical acquisition protocols across all scanners; however, we acknowledge that this may not always be possible, especially when including different scanners across vendors. Future studies should address the contribution of different acquisition factors (e.g., number of gradient directions, voxel resolution) one by one while controlling for any other factors.

This study assesses the reproducibility of DTI and NODDI parameters in four major WM tracts. The choice of the structures is motivated by both the availability of the atlases offered by TractFinder (Young et al. 2024) at the time when the study was conducted, and by the clinical significance of these selected tracts. Future work should assess the reproducibility of diffusion parameters in other brain structures, especially in grey matter regions, as well as in pathological conditions, for example, brain tumours.

## 5 Conclusions

We present a reproducibility analysis for DTI‐derived FA and MD and NODDI‐derived ODI, NDI and FWF in four clinically relevant WM tracts measured across three scanners of the same model. Our results provide evidence that FA and MD have excellent reproducibility (ICC ≥ 0.964) across scanners of the same model, when using identical acquisition schemes, pre‐processing pipelines and automatically segmented ROIs. NODDI ODI and NDI also have excellent reproducibility (ICC ≥ 0.911). However, caution is advised when using FWF, as statistically significant differences were detected in the left IFOF and OR. Given that the same cohort was studied across the scanner, that should not be the case.

Determining inter‐scanner reproducibility of MRI‐derived parameters in healthy subjects provides reference values that are crucial in planning future multi‐centre studies. The level of reproducibility and variability of the diffusion parameters reported here can aid future QIB studies which use inter‐scanner data to discern whether the observed change in the parameter is biologically meaningful or to what extent it is significantly contaminated with non‐biological measurement variability.

## Funding

The work is supported by the University College London (UCL) and the National Physical Laboratory (NPL) as part of the EPSRC iCASE 2021–22 programme. The NPL contribution was funded by the Department for Science, Innovation and Technology (formerly Department of Business, Engineering and Industrial Strategy) through the National Measurement System under the Data Science Modelling & Analytics Applications theme. In addition, all research at Great Ormond Street Hospital NHS Foundation Trust and UCL Great Ormond Street Institute of Child Health is made possible by the NIHR Great Ormond Street Hospital Biomedical Research Centre.

## Ethics Statement

The data for this study was acquired across three centres: UCL GOS Institute of Child Health and Great Ormond Street Hospital for Children (datasets labelled UCL), MRC Cognition and Brain Sciences Unit, University of Cambridge (datasets labelled CAM) and CUBRIC, School of Psychology, Cardiff University (datasets labelled CAR). Ethical approval (number 2780/001) for this study was granted by the UCL Research Ethics Committee (REC).

## Consent

The authors have nothing to report.

## Conflicts of Interest

Jonathan D. Clayden and Chris A. Clark are inventors on pending US and European patents which cover the core Tractfinder technology. The other authors declare no conflicts of interest.

## Data Availability

Research data are not shared. Pre‐processing pipeline script available: https://github.com/aga‐sierhej/inter_scanner_reproducibility/tree/main.

## Appendix A

**TABLE A1:** Mean and SD of DTI parameters (FA and MD) for each centre across the subject.

	DTI															
	Left								Right							
	CST		AF		IFOF		OR		CST		AF		IFOF		OR	
	Mean	±SD	Mean	±SD	Mean	±SD	Mean	±SD	Mean	±SD	Mean	±SD	Mean	±SD	Mean	±SD
FA																
CAM	0.477	0.149	0.473	0.120	0.478	0.133	0.490	0.144	0.481	0.148	0.457	0.114	0.480	0.141	0.483	0.125
CAR	0.483	0.150	0.471	0.120	0.479	0.132	0.489	0.143	0.486	0.151	0.457	0.115	0.481	0.139	0.484	0.124
UCL	0.486	0.149	0.476	0.119	0.483	0.133	0.495	0.144	0.488	0.149	0.460	0.115	0.483	0.141	0.485	0.125
Mean of scanners	0.482	0.149	0.473	0.120	0.480	0.133	0.491	0.144	0.485	0.149	0.458	0.115	0.481	0.140	0.484	0.125
SD of scanners	0.003742	0.000471	0.002055	0.000471	0.002160	0.000471	0.002625	0.000471	0.002944	0.001247	0.001414	0.000471	0.001247	0.000943	0.000816	0.000471
CoV	0.78%	0.32%	0.43%	0.39%	0.45%	0.36%	0.53%	0.33%	0.61%	0.84%	0.31%	0.41%	0.26%	0.67%	0.17%	0.38%
MD^a^																
CAM	0.576	0.043	0.593	0.037	0.621	0.045	0.628	0.058	0.577	0.044	0.590	0.035	0.622	0.042	0.625	0.056
CAR	0.573	0.042	0.592	0.037	0.619	0.043	0.625	0.056	0.572	0.044	0.586	0.035	0.618	0.040	0.622	0.055
UCL	0.580	0.042	0.597	0.036	0.624	0.045	0.631	0.057	0.581	0.045	0.590	0.034	0.621	0.042	0.626	0.055
Mean of scanners	0.576	0.042	0.594	0.037	0.621	0.044	0.628	0.057	0.577	0.044	0.589	0.035	0.620	0.041	0.624	0.055
SD of scanners	0.002867	0.000471	0.002160	0.000471	0.002055	0.000943	0.002449	0.000816	0.003682	0.000471	0.001886	0.000471	0.001700	0.000943	0.001700	0.000471
CoV	0.50%	1.11%	0.36%	1.29%	0.33%	2.13%	0.39%	1.43%	0.64%	1.06%	0.32%	1.36%	0.27%	2.28%	0.27%	0.85%

*Note:* Mean and SD deviation of scanners is included. CoV between the three centre is calculated for each tract.

^a^ Unit: $\times 10^{-3}\frac{{\mathrm{mm}}^{2}}{s}$.

**TABLE A2:** Mean and SD of NODDI parameters (ODI, NDI, FWF) for each centre across the subject.

	NODDI															
	Left								Right							
	CST		AF		IFOF		OR		CST		AF		IFOF		OR	
	Mean	±SD	Mean	±SD	Mean	±SD	Mean	±SD	Mean	±SD	Mean	±SD	Mean	±SD	Mean	±SD
ODI																
CAM	0.254	0.109	0.252	0.091	0.235	0.103	0.229	0.114	0.251	0.109	0.261	0.088	0.231	0.095	0.233	0.112
CAR	0.252	0.108	0.253	0.091	0.235	0.102	0.23	0.113	0.25	0.109	0.263	0.089	0.232	0.094	0.233	0.109
UCL	0.247	0.107	0.249	0.089	0.231	0.102	0.225	0.112	0.245	0.107	0.259	0.088	0.23	0.094	0.232	0.11
Mean of scanners	0.251	0.108	0.251	0.090	0.234	0.102	0.228	0.113	0.249	0.108	0.261	0.088	0.231	0.094	0.233	0.110
SD of scanners	0.002944	0.000816	0.001700	0.000943	0.001886	0.000471	0.002160	0.000816	0.002625	0.000943	0.001633	0.000471	0.000816	0.000471	0.000471	0.001247
CoV	1.17%	0.76%	0.68%	1.04%	0.81%	0.46%	0.95%	0.72%	1.06%	0.87%	0.63%	0.53%	0.35%	0.50%	0.20%	1.13%
NDI																
CAM	0.641	0.074	0.609	0.063	0.575	0.052	0.575	0.057	0.638	0.071	0.607	0.061	0.567	0.049	0.569	0.055
CAR	0.645	0.074	0.608	0.063	0.574	0.052	0.573	0.057	0.645	0.073	0.611	0.062	0.572	0.049	0.573	0.056
UCL	0.645	0.078	0.612	0.065	0.579	0.055	0.58	0.06	0.642	0.077	0.613	0.062	0.576	0.052	0.576	0.058
Mean of scanners	0.644	0.075	0.610	0.064	0.576	0.053	0.576	0.058	0.642	0.074	0.610	0.062	0.572	0.050	0.573	0.056
SD of scanners	0.001886	0.001886	0.001700	0.000943	0.002160	0.001414	0.002944	0.001414	0.002867	0.002494	0.002494	0.000471	0.003682	0.001414	0.002867	0.001247
CoV	0.29%	2.50%	0.28%	1.48%	0.38%	2.67%	0.51%	2.44%	0.45%	3.39%	0.41%	0.76%	0.64%	2.83%	0.50%	2.21%
FWF																
CAM	0.067	0.061	0.058	0.047	0.056	0.051	0.061	0.06	0.065	0.06	0.054	0.044	0.048	0.047	0.051	0.054
CAR	0.067	0.06	0.054	0.045	0.052	0.048	0.056	0.054	0.065	0.059	0.055	0.044	0.049	0.047	0.054	0.055
UCL	0.076	0.063	0.067	0.049	0.065	0.053	0.071	0.06	0.074	0.063	0.062	0.046	0.058	0.049	0.063	0.057
Mean of scanners	0.070	0.061	0.060	0.047	0.058	0.051	0.063	0.058	0.068	0.061	0.057	0.045	0.052	0.048	0.056	0.055
SD of scanners	0.004243	0.001247	0.005437	0.001633	0.005437	0.002055	0.006236	0.002828	0.004243	0.001700	0.003559	0.000943	0.004497	0.000943	0.005099	0.001247
CoV	6.06%	2.03%	9.11%	3.47%	9.43%	4.06%	9.95%	4.88%	6.24%	2.80%	6.24%	2.11%	8.70%	1.98%	9.11%	2.25%

*Note:* Mean and SD deviation of scanners is included. CoV between the three centres is calculated for each tract.
